# A biologist, a statistician, and a bioinformatician walk into a conference room… and walk out with a great metagenomics project plan

**DOI:** 10.3389/fpls.2014.00250

**Published:** 2014-06-03

**Authors:** Ann E. Stapleton

**Affiliations:** Biology and Marine Biology, University of North Carolina WilmingtonWilmington, NC, USA

**Keywords:** computational resources, metagenomes, metagenomic experimental design, metadata, statistical analysis, optimal experimental design, multivariate data analysis

Reviews of metagenomics analysis often emphasize the interdisciplinary and technical aspects of data analysis (Knief, 2014; Sharpton, 2014). How might these recommendations be implemented for future projects? In this opinion, I provide some areas to consider, especially in experimental design and full-data-cycle planning, and in expertise areas of value to metagenomics projects. This opinion is structured as a hypothetical conversation that reviews state-of-the art in these areas and brings out the various aspects of metagenomics project design.

Let's consider an example project in plant-microbe metagenomics—analysis of microbial metagenome functional genes that predict plant yield differences in a horticultural crop species. I've framed a discussion of key points as a conversation, perhaps at the third or fourth meeting, after participants have described their range of expertise.

***Biologist:*** We are here today because we're all interested in doing a great research project in the rapidly growing area of metagenomics. We've heard about this in human biology and health (Human Microbiome Project Consortium, 2012; Morgan et al., 2013), now we'd like to be sure we think through the research aspects for crop biological systems. Let's consider some biological characteristics, such as homeostasis—resilience to disturbance—and adaptation, as general background. Homeostasis, or robustness, is the ability to respond transiently, and then go back to something that functions like the original measured state. In biology, we usually talk about this in the simplest examples using an X-Y line graph with a peak (Calabrese and Blain, 2005; Paine et al., 2012). For example, responses to plant hormones often show a peak at a certain concentration (Taiz and Zeiger, 2006). For communities of organisms, this is often described as ecological resilience and may be measured at multiple levels of organization. We'd like to understand if resilience is happening and if it is important.

***Statistician:*** There are some interesting statistical implications for defining your important questions as curves. Let's relate this to recent “design-of-experiment” research, which is about how to create the most efficient experiment. For curves, you will need to think about how few points can be used to fit such curves (you will need several amounts from your X and Y axes), and how the replicates should be arranged… for example, should there be more replicates on the steep sections of a curve or at the tails. This is an area of research called response surface design. Current approaches in this field include low-dimensional Bayesian (Ryan et al., 2014) and Gaussian models (Harari and Steinberg, 2013).

***Biologist:*** Another biological aspect to consider is adaption, the ability to detect a stimulus after the system stabilized, which is usually graphically illustrated as a step-shaped X-Y plot, with the adaptive process happening in the “step” phase, with the response in the “riser” sections (Lim et al., 2013). So, in my particular plant-microbe research area/model system, I am interested in analyzing metagenome changes that can capture these patterns and determine if they are different in low-yield and high-yield plants.

***Statistician:*** Another applied statistical topic is the effect of assumptions behind various analysis methods, from more classical assumptions of normality to choosing a specific possible distribution as a Bayesian prior. This is especially important to consider as a metagenomic sample is highly multivariate (there are many gene sequences within each sample), and underlying assumptions about distributions will constrain what you can reliably detect. There can be useful information for understanding your biological system in the higher-order correlation and autocorrelation (Gallagher et al., 2014) within the samples, so it is worth spending time thinking about how to incorporate what you already know about your system into your analysis choices.

***Bioinformatician:*** It does no good to have data that you can't analyze in a reasonable time frame! We will need to plan for storage of the raw data and feeding of the raw data into the quality control programs (Knight et al., 2012). How much data and how complex is the analysis going to be?

***Statistician:*** It's quite a balancing act to determine the number of samples. We will need to ensure that we have the resources to do a careful walk-through and thorough testing of the data analysis, with the same seriousness we would use for pilot tests of lab procedures, for various options. For example, we should locate any existing known-truth data and develop software code to produce known-truth data—this is where we embed a known pattern, such as a particular gene present in large amounts, in a background of other genes. This known-truth generation process is usually called simulation in statistics. We would also want to use the most similar already-available real data for testing of our analysis methods. We want to know as much as possible about accuracy and precision before beginning the experimental data collection.

***Biologist:*** I am hearing that we need to focus the question or we will have a huge number of samples. What kinds of pilot tests can we do that would help us keep the sample numbers low but have the maximum power to make predictions?

***Bioinformatician:*** For processing raw reads before doing statistical tests, we will need to test the options for quality control processing (the parameters); it's important to understand how these work before selecting ranges to test, to avoid wasting time testing things that don't affect the output much and to define how some parameter choices depend on other parameter choices (Zhou and Rokas, 2014). This is a place where the known-truth simulations that were mentioned can be helpful. We will also want to track the current best practice in the field using listservs and web resources (Li et al., 2012), as optimal methods can be updated very quickly.

***Statistician:*** We would want to leverage the multivariate aspects of the data for statistical comparison. Typically I would use R packages for this and I'd like your opinion on the computational feasibility. I would also like more details on pre-processing—how extensive is the data cleaning?

***Bioinformatician:*** We would want to assemble sequences from the reads that come from the sequencing machine to reduce the error and increase the information in each “sequence unit,” but there is no single best assembly method; using combinations of methods will increase the computational demand substantially. Another important computational consideration is minimizing the trafficking across the network and doing data transfers efficiently. With large numbers of large samples, we will need to use efficient code for data analysis. If the analysis code is written in R, we need to ensure that certain key parts are in C, determine if high performance computing resources are needed and how most easily access those resources. Running statistical R code on a computing cluster or the national XSEDE resource does not guarantee speedup, so we would need to figure out how to optimize the analysis enough to finish it in an acceptable length of time. Another consideration is how to determine how many times the analysis will be tested/re-run, to decide how to organize the code for re-use.

***Biologist:*** Another aspect of metagenomic sequence data is that it can be considered at multiple levels, with annotations of function that come from sources ranging from ontologies (Ashburner et al., 2000) to literature citations (Raychaudhuri et al., 2009), and can be placed in groups ranging from one annotation per sequence to one annotation category that includes thousands of sub-category sequences.

***Statistician:*** Multilevel, or hierarchical, models can be used to handle data labels that have subgroups like the GO annotations, but they can be computationally challenging to fit. We will need to consider these ways of labeling groups and the resulting constraints on comparing samples as we test different analysis methods, in order to choose models that can handle these types of graphs. Different levels of nesting, correlation and comparisons of sets from different parts of an acyclic graph present challenges, for example (Tryputsen et al., 2014).

***Biologist:*** Let me summarize what I see as the dimensions of data analysis we are considering… experimental design tradeoffs, quality control, model fit, assumptions, and their interactions and dependencies. This certainly requires true collaboration, and we should think about formalizing what we've discussed in high-level systems modeling tools http://insightmaker.com/, (North et al., 2013) to explore the costs and benefits and thus optimize our experimental plan.

***Statistician:*** This kind of high-level modeling is sometimes called decision support, and it certainly could help us convince ourselves and our reviewers and colleagues that we have the best possible experimental plan. We do seem to have a good start on synthesis across our different fields from this conversion and these suggestions.

***Bioinformatician:*** We also need to consider metadata, storage, and classroom or citizen use—it's not just the publication, it's the impact, the reuse as well as citations (Piwowar and Vision, 2013; Roche et al., 2014). In fact, there are people who specialize in this—let's add an information science librarian to the mix to advise us on curation (Whyte and Allard, 2014). Now that all the pieces of a great project are on the table, the whiteboard, and the shared computer files, we can think more about the details for our next project meeting, and we have an excellent background to do superb metagenomic science.

This conversation highlights current recommendations and considerations for efficient metagenomics data collection and data analysis. I recommend that project teams consider these general topic areas and involve experts in all these areas when they next develop project plans.

## Conflict of interest statement

The author declares that the research was conducted in the absence of any commercial or financial relationships that could be construed as a potential conflict of interest.
